# Supplementary material for: Manifestations of metastable criticality in the long-range structure of model water glasses
Source: Nat Commun. 2021 Jun 7;12:3398. doi: 10.1038/s41467-021-23639-2 (PMC8185069; doi:10.1038/s41467-021-23639-2)
Supplement: Supplementary file 1 — Supplementary Information [file 41467_2021_23639_MOESM1_ESM.pdf]

Submitted to *Nature Communications*

Supplementary Information for:

Manifestations of metastable criticality in the long-range structure of model water glasses

Thomas E. Gartner III[a], Salvatore Torquato[a,b,c,d], Roberto Car[a,b,c,d], and Pablo G. Debenedetti[e,*]

[a]Department of Chemistry, Princeton University, Princeton, NJ 08544;

[b]Department of Physics, Princeton University, Princeton, NJ 08544;

[c]Program in Applied and Computational Mathematics, Princeton University, Princeton, NJ 08544;

[d]Princeton Institute for the Science and Technology of Materials, Princeton University, Princeton, NJ 08544;

[e]Department of Chemical and Biological Engineering, Princeton University, Princeton, NJ 08544

[*]Corresponding author: pdebene@princeton.edu

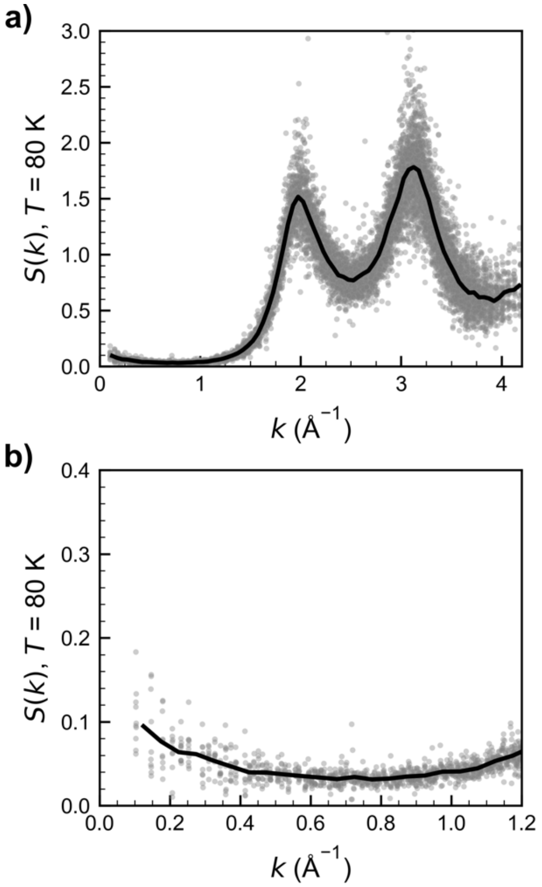

Supplementary Figure 1: (a,b) Static structure factor $S(k)$ as a function of wavenumber k in the TIP4P/2005 glass at temperature T = 80 K after isobaric cooling at pressure P = 1860 bar and a cooling rate q_T = -1.0 K/ns. Grey circles are the raw $S(k)$ values obtained via main text Equation 1 from the 10 independent glass configurations, and the black line is an average $S(k)$ obtained by taking the mean of all raw $S(k)$ values that fall within an interval Δk = 0.05 Å^{-1}. Panel (b) is the same data as (a) plotted on a different axis scale to focus on the low-k region.

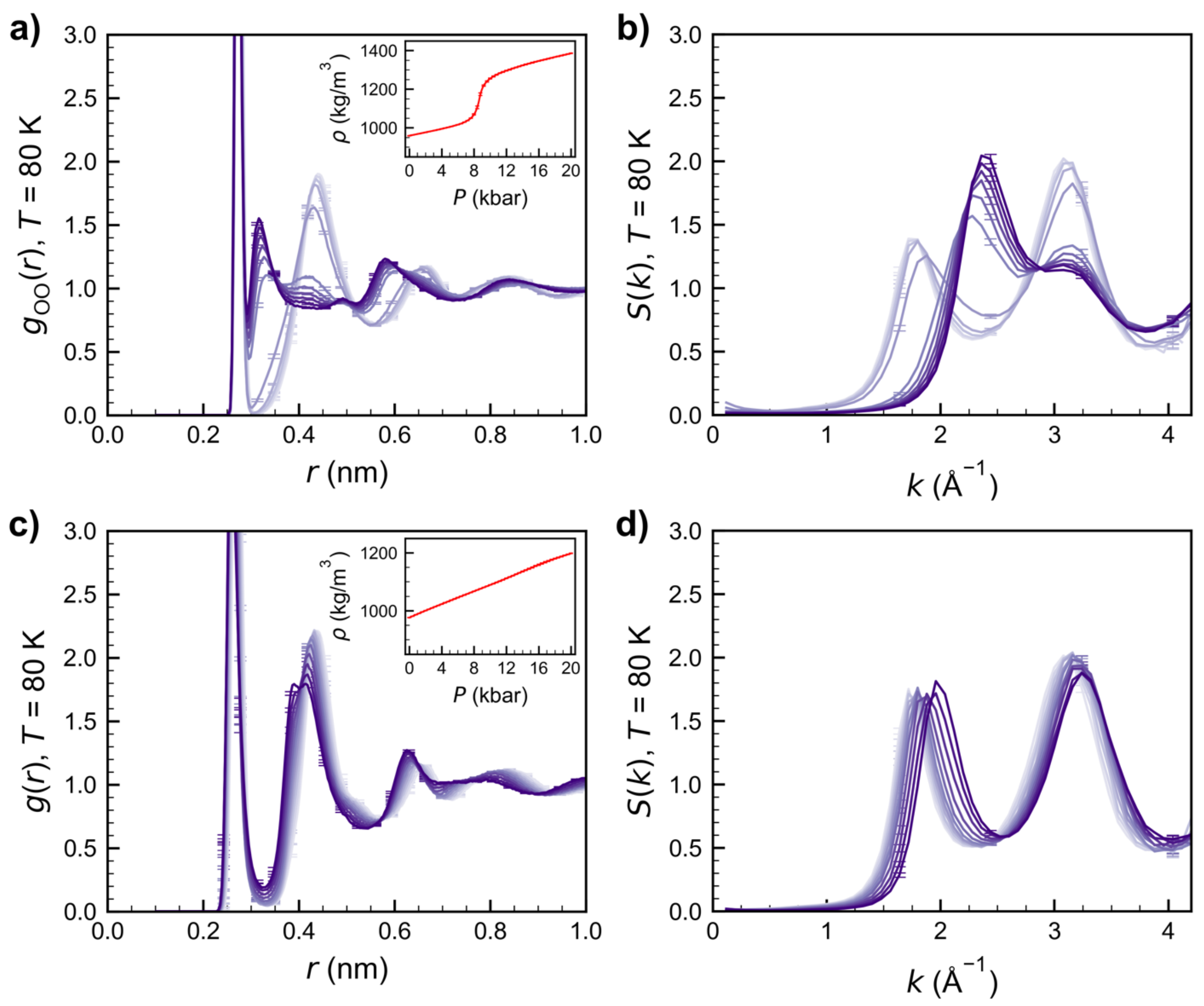

Supplementary Figure 2: Pressure induced LDA→HDA transition at T = 80 K. Main plots are the (a) oxygen-oxygen radial distribution function ($g_{OO}(r)$) or (b) $S(k)$ in TIP4P/2005, or (c) the total radial distribution function ($g(r)$) or (d) $S(k)$ in mW, plotted from P = 1 bar (lightest purple) to P = 20,000 bar (darkest purple) in steps of 1,000 bar along a pressurization ramp. Insets in (a) and (c) are the mass density as a function of pressure. Error bars represent 95% confidence intervals obtained from the standard error of the mean of 10 independent trials.

Supplementary Note 1:

To explore the pressurization-induced LDA→HDA transition in TIP4P/2005 and mW, we took the final LDA configurations obtained by isobaric cooling at P = 1 bar and q_T = -10 K/ns and performed simulations with a stepwise increase in pressure from 1 bar to 20,000 bar in steps of 100 bar at a pressurization rate of q_P = 100 bar/ns. As seen previously,[1] in TIP4P/2005 (Supplementary Figure 2a-b) upon pressurization we observed a first-order-like phase transition from LDA to HDA in the vicinity of P = 8000-9000 bar, visible in both the sharp increase in density and significant change in the $g_{OO}(r)$ and $S(k)$ from LDA-like to HDA-like local structures.[2-6] By contrast, while mW (Supplementary Figure 2c-d) does exhibit polyamorphism,[7] the transition from LDA to HDA was gradual and with a less significant change in local structure.

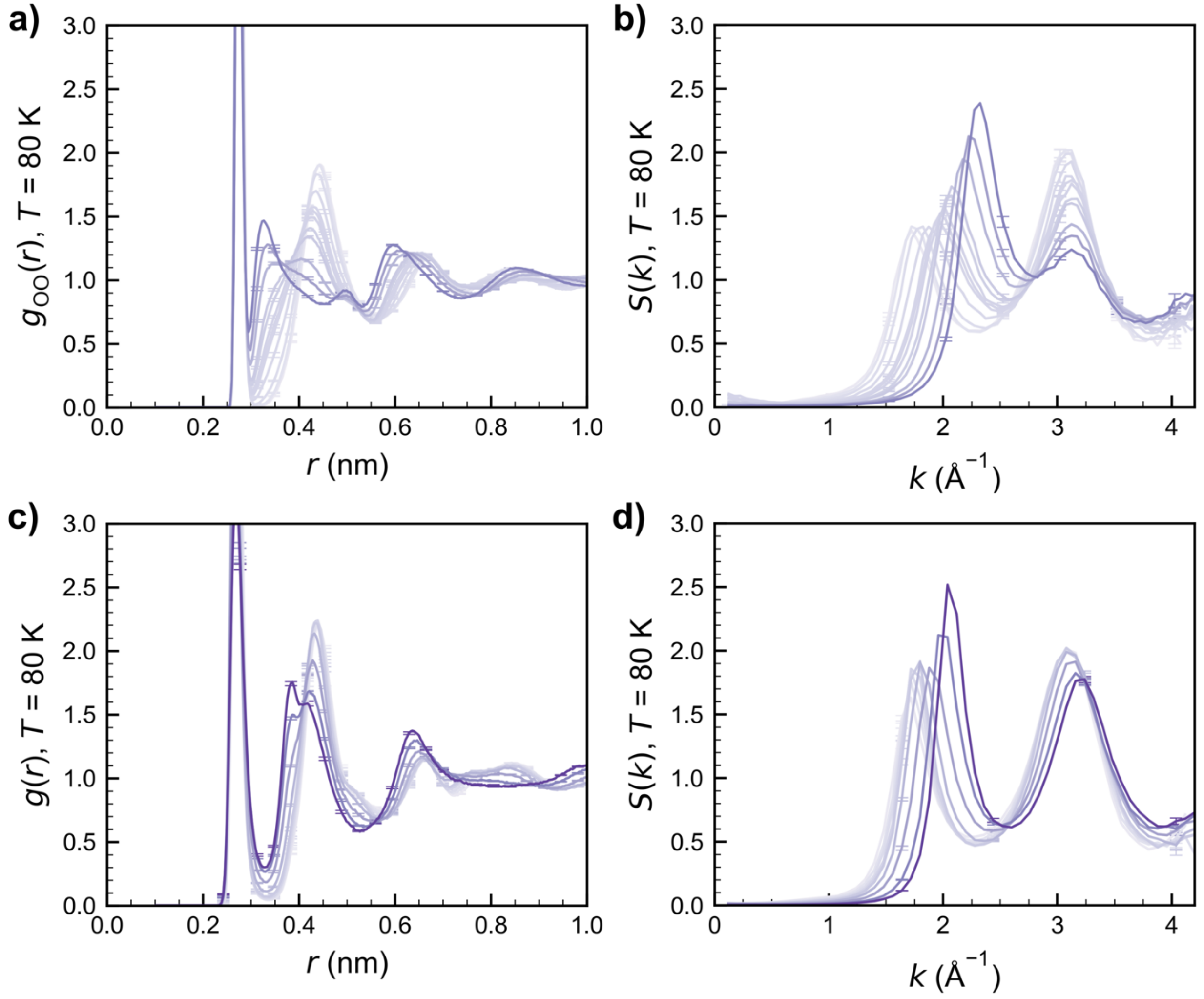

Supplementary Figure 3: Structure of amorphous ices at T = 80 K prepared via isobaric cooling. (a) $g_{OO}(r)$ or (b) $S(k)$ in TIP4P/2005 cooled at a cooling rate of q_T = -1.0 K/ns and (c) $g(r)$ or (d) $S(k)$ in mW cooled at q_T = -10 K/ns. In all panels, darker purple denotes higher pressure, ranging from (a,b) P = -1.0, 0.001, 1.0, 1.5, 1.75, 1.86, 2.0, 2.5, 3.0, 5.0, 7.0, and 10.0 kbar, and (c,d) P = -1.0, 0.001, 1.0, 2.0, 3.0, 5.0, 7.0, 10.0, and 15.0 kbar. Error bars represent 95% confidence intervals obtained from the standard error of the mean of 10 independent trials.

Supplementary Note 2:

In both TIP4P/2005 and mW, the structure of amorphous ices prepared isobarically at low and high pressures showed characteristics of LDA and HDA ice, respectively. However, in contrast to the sharp LDA/HDA transition seen upon pressurization of TIP4P/2005 at T = 80 K (Supplementary Figure 2a-b), amorphous ices prepared by isobaric quenching at intermediate pressures showed a combination of LDA-like and HDA-like local structures, as evidenced by the gradual change in $g_{OO}(r)$ and $S(k)$ as a function of pressure (Supplementary Figure 3a-b). Interestingly, for both TIP4P/2005 and mW, the pressures at which signatures of both LDA and HDA are visible in the local structures shown in Supplementary Figure 3 are the same pressures at which the locus of the glass transition temperature (T_g) as a function of pressure (main text Figure 3) transitioned from anomalous negative slope to simple liquid-like positive slope.

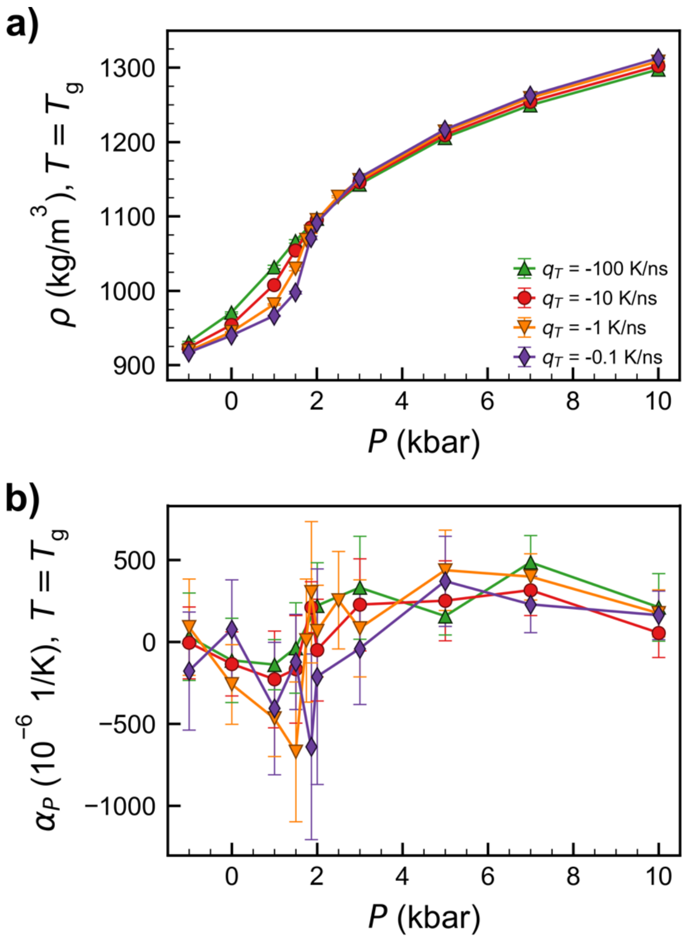

Supplementary Figure 4: Fluid properties in TIP4P/2005 at the glass transition temperature (T_g). (a) Mass density, ρ, and (b) coefficient of thermal expansion, α_P, as a function of pressure (P) at the T_g shown in main text Figure 3a. Colors and symbols denote different cooling rates (q_T) as marked, and error bars are 95% confidence intervals obtained from the standard error of the mean of 10 trials.

Supplementary Note 3:

The mass density and coefficient of thermal expansion showed interesting anomalous behavior at pressures below the liquid-liquid critical pressure for TIP4P/2005 (P_c = 1861 bar).[8] As the cooling rate decreased, the density at T_g (Supplementary Figure 4a) developed an inflection point, and the slope at the inflection point increased sharply with decreasing q_T. Extrapolating this behavior to infinitely slow cooling (i.e., equilibrium), one might expect the inflection point to become the discontinuous first-order transition between high- and low-density liquids. At high pressures, the densities at T_g were largely independent of cooling rate. The coefficient of thermal expansion (Supplementary Figure 4a), calculated by numerically evaluating $\alpha_P = \frac{1}{V}\left(\frac{\partial V}{\partial T}\right)_P$ along the cooling trajectories, showed anomalous negative values at T_g for pressures below P_c, which transitioned to positive values at high pressure. Interestingly, for the two quantities shown here, as well as the T_g vs. P curves shown in main text Figure 3, the anomalous behavior was observed for $P < P_c$ (i.e., in the LDL or LDA states). We plan to explore these trends in more detail in future work.

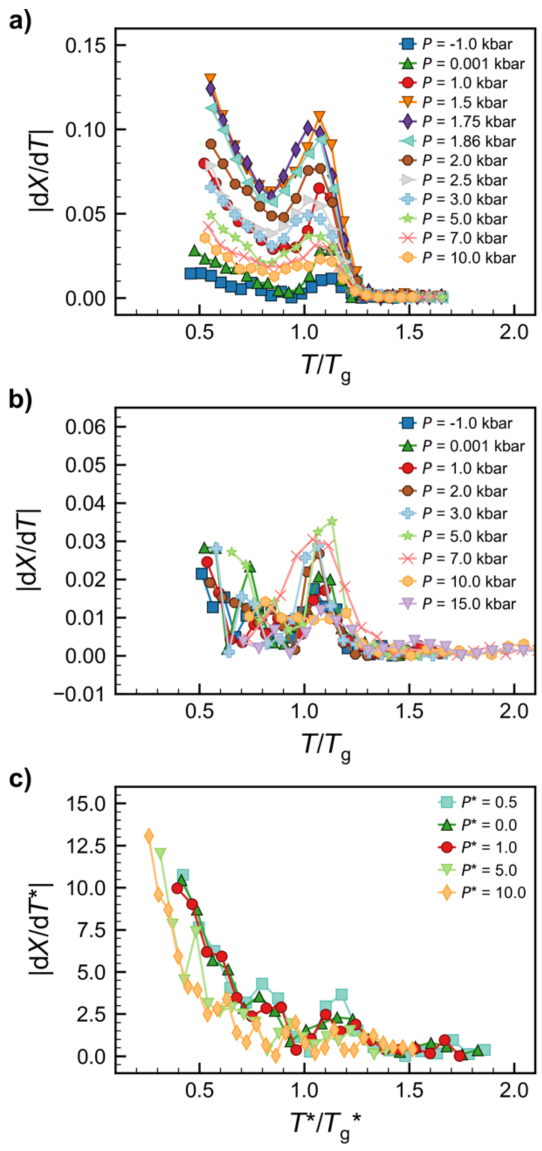

Supplementary Figure 5: Magnitude of the temperature derivative of the non-equilibrium index ($|dX/dT|$) as a function of T (normalized by T_g) for isobaric cooling of (a) TIP4P/2005 at a cooling rate q_T = -1.0 K/ns, (b) mW at q_T = -10 K/ns, and (c) Kob-Andersen mixture at $q_T = -5.55 \times 10^{-6}\ \tau^{-1}$. Colors and symbols denote different pressures as marked.

Supplementary Note 4:

The temperature derivative of the non-equilibrium index (dX/dT, calculated by numerical differentiation of the data presented in main text Figure 5) reveals interesting pressure- and temperature-dependent trends in X for water-like models. For both TIP4P/2005 (Supplementary Figure 5a) and mW (Supplementary Figure 5b), X exhibits an increase in slope magnitude at temperatures just above the glass transition temperature T_g, followed by a regime of slower change in X just below T_g, and finally followed by an additional increase in the rate of change of X at the lowest temperatures. By contrast, clearly identifiable regimes are absent in the Kob-Andersen mixture (Supplementary Figure 5c). Furthermore, the increases/decreases in dX/dT in TIP4P/2005 are most pronounced near the critical pressure, possibly illustrating the impact of critical slowing

down.[9] In future work, we plan to map in detail the changes in $S(k)$, isothermal compressibility, and/or density as a function of (T, P) to identify the source of these different regimes in dX/dT for water-like models.

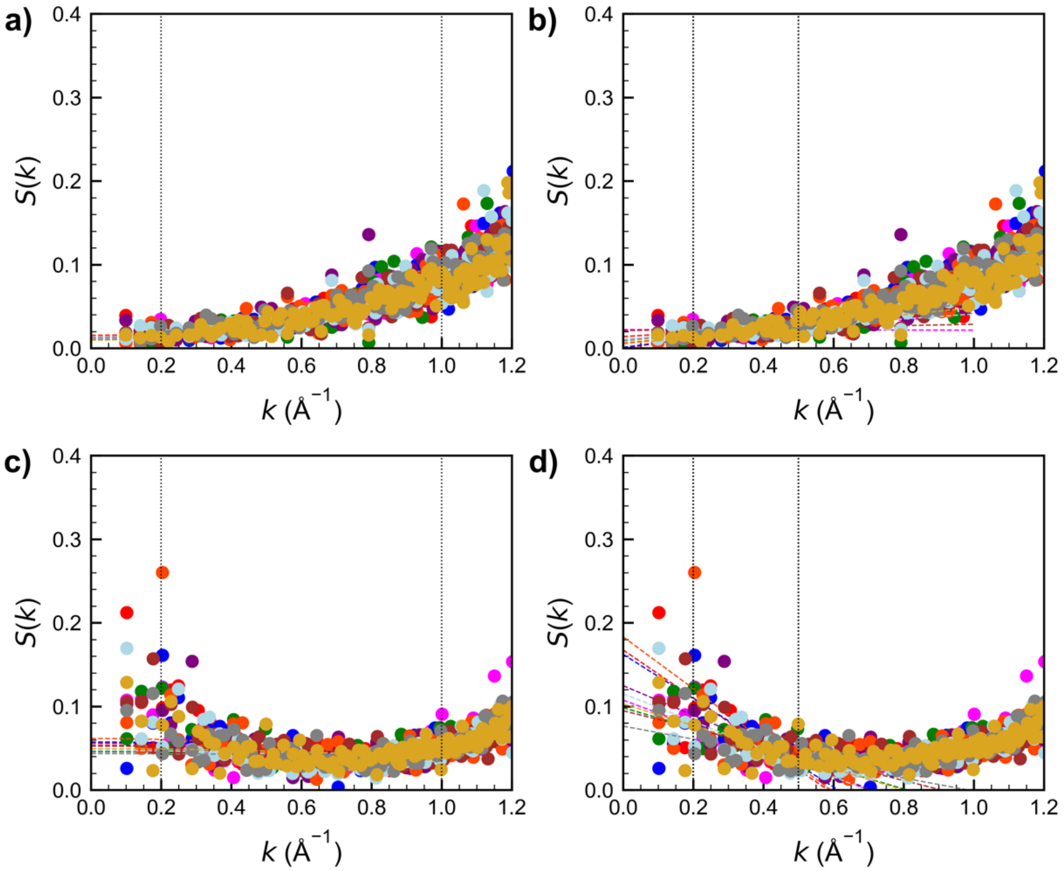

Supplementary Figure 6: Examples of static structure factor ($S(k)$) fitting and extrapolation procedure for TIP4P/2005 glasses at T = 80 K prepared by isobaric quenching at (a,b) P = 1 bar and (c,d) P = 1500 bar cooled at q_T = -1.0 K/ns. Colored circles denote $S(k)$ calculated from 10 independent trials, vertical black dashes lines denote the range of k over which the fits were performed, and colorful dashed lines represent the extrapolation to low-k for each configuration. In (a,c), extrapolation to zero wavenumber was performed by fitting $S(k)$ to a quadratic function $y = c_2k^2 + c_0$ over the range 0.2 A^{-1} ≤ k ≤ 1.0 A^{-1}, and in (b,d) extrapolation was performed by fitting $S(k)$ to a linear function $y = c_1k + c_0$ over the range 0.2 A^{-1} ≤ k ≤ 0.5 A^{-1}.

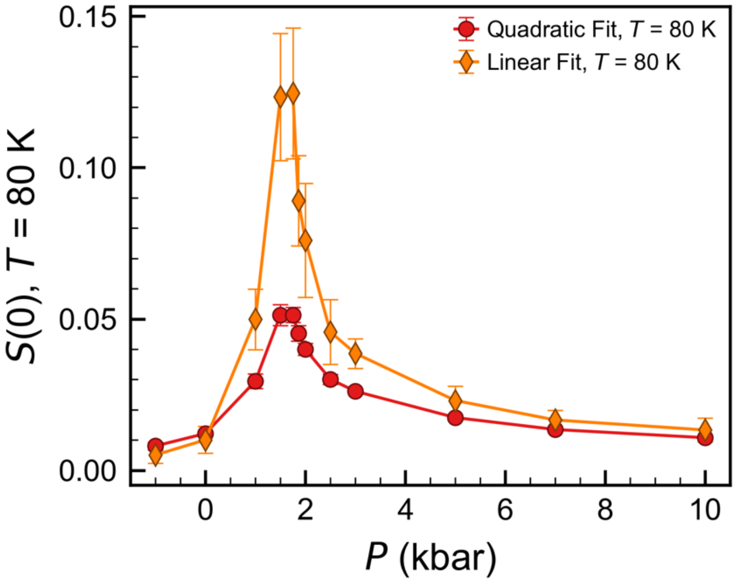

Supplementary Figure 7: Zero-wavenumber limit of the static structure factor as a function of pressure in TIP4P/2005 glasses quenched isobarically to $T = 80$ at various pressures and a cooling rate of q_T = -1.0 K/ns. Colors and symbols denote different low-k extrapolation procedures as marked. Error bars represent 95% confidence intervals obtained from the standard error of the mean over 10 different trials.

Supplementary Note 5:
As seen in Supplementary Figures 6 and 7, in TIP4P/2005 glasses prepared near the liquid-liquid critical pressure of P_c = 1861 bar,[8] the $S(k)$ exhibited an upturn in low-k (e.g., P = 1500 bar results in Supplementary Figure S6c-d). However, whether we performed a quadratic or linear fit to $S(k)$ to extrapolate to $k = 0$, the overall trend in $S(0)$ vs. P was preserved (Supplementary Figure 7), as the peak in $S(0)$ occurred at the same pressure, and the low- and high-P limits agreed within error. As discussed in the main text, the quadratic fit to $S(k)$ may underpredict the value of $S(0)$ for pressures near the LLCP due to the appearance of anomalous critical fluctuations, however given the results in Supplementary Figure 7 we decided to report the quadradic fit in the main text due to its decreased sensitivity to numerical noise in the low-k data. We also tested the addition of a Lorentzian term in the fit to capture the anomalous critical scattering contribution as used in Ref. [8]; this approach resulted in similar $S(0)$ results to the linear fit shown above. We also emphasize that we did not observe any evidence of a low-k upturn in the mW results for any condition, supporting the conclusion that mW does not exhibit an LLCP.

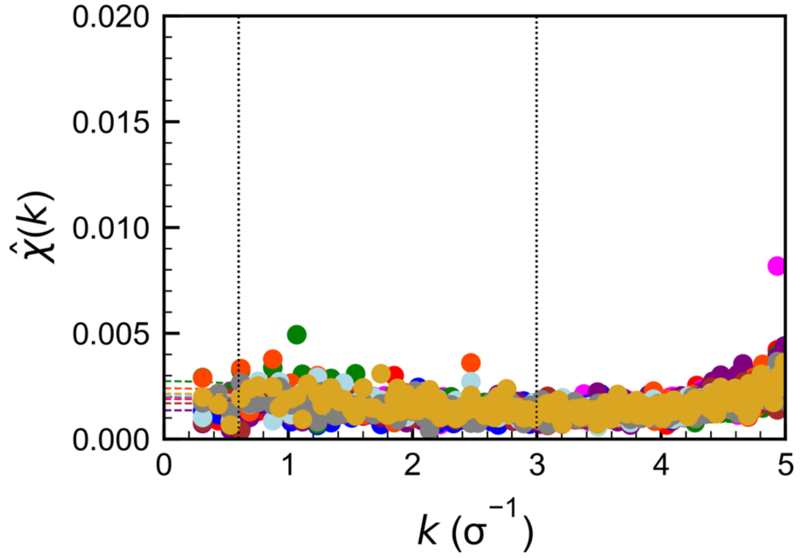

Supplementary Figure 8: Examples of spectral density ($\hat{\chi}(k)$) fitting and extrapolation procedure for Kob-Andersen glasses at T^* = 0.1 prepared by isobaric quenching at P^* = 0 cooled at q_T = -5.55 x 10^{-6} τ^{-1}. Colored circles denote $\hat{\chi}(k)$ calculated from 10 independent trials, vertical black dashed lines denote the range of k over which the fits were performed, and colorful dashed lines represent the extrapolation to low-k for each configuration. Extrapolation to zero wavenumber was performed by fitting $\hat{\chi}(k)$ to a 4th-order polynomial $y = c_4k^4 + c_2k^2 + c_0$ over the range 0.6 $\sigma^{-1} \leq k \leq$ 3.0 σ^{-1}.

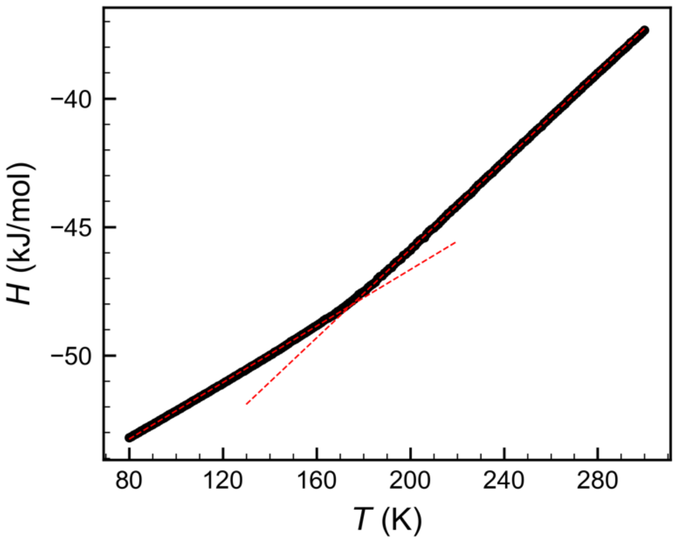

Supplementary Figure 9: Enthalpy (*H*) as a function of temperature along an isobaric cooling ramp (black symbols) for TIP4P/2005 at P = 1860 bar and q_T = -1.0 K/ns. We defined the glass transition temperature T_g reported in main text Figure 3 as the intersection of lines fit to the high- and low-temperature branches of the *H* vs. *T* curves (red dashed lines).

Supplementary References:

1. Martelli, F., Torquato, S., Giovambattista, N. & Car, R. Large-Scale Structure and Hyperuniformity of Amorphous Ices. *Phys. Rev. Lett.* **119**, 136002 (2017).
2. Mariedahl, D. *et al.* X-ray Scattering and O-O Pair-Distribution Functions of Amorphous Ices. *J. Phys. Chem. B* **122**, 7616-7624 (2018).
3. Amann-Winkel, K. *et al.* Colloquium: Water's controversial glass transitions. *Rev. Mod. Phys.* **88**, 011002 (2016).
4. Engstler, J. & Giovambattista, N. Heating-and pressure-induced transformations in amorphous and hexagonal ice: A computer simulation study using the TIP4P/2005 model. *J. Chem. Phys.* **147**, 074505 (2017).
5. Perakis, F. *et al.* Diffusive dynamics during the high-to-low density transition in amorphous ice. *Proc. Natl. Acad. Sci. U.S.A.* **114**, 8193-8198 (2017).
6. Shi, R. & Tanaka, H. Direct Evidence in the Scattering Function for the Coexistence of Two Types of Local Structures in Liquid Water. *J. Am. Chem. Soc.* **142**, 2868-2875 (2020).
7. Limmer, D. T. & Chandler, D. Theory of amorphous ices. *Proc. Natl. Acad. Sci. U.S.A.* **111**, 9413-9418 (2014).
8. Debenedetti, P. G., Sciortino, F. & Zerze, G. H. Second Critical Point in Two Realistic Models of Water. *Science* **369**, 289-292 (2020).
9. Hohenberg, P. C. & Halperin, B. I. Theory Of Dynamic Critical Phenomena. *Rev. Mod. Phys.* **49**, 435-479 (1977).
